# Laboratory diagnosis of human T-lymphotropic virus in Brazil: assays, flowcharts, challenges, and perspectives

**DOI:** 10.1590/0037-8682-0175-2021

**Published:** 2021-06-02

**Authors:** Adele Caterino-de-Araujo, Edel Figueiredo Barbosa-Stancioli, José Boullosa Alonso, Mayra Gonçalves Aragón, Bernardo Galvão-Castro, Ricardo Ishak, Carolina Rosadas

**Affiliations:** 1 Instituto Adolfo Lutz, Centro de Imunologia, Laboratório de Pesquisa em HTLV, São Paulo, SP, Brasil.; 2 Universidade Federal de Minas Gerais, Instituto de Ciências Biológicas, Laboratório de Virologia Básica e Aplicada, Belo Horizonte, MG, Brasil.; 3 Ministério da Saúde, Secretaria de Vigilância em Saúde, Departamento de Doenças de Condições Crônicas e Infecções Sexualmente Transmissíveis, Brasília, DF, Brasil.; 4 Universidade Federal do Espírito Santo, Programa de Pós-Graduação em Doenças Infecciosas, Vitória, ES, Brasil.; 5 Escola Bahiana de Medicina e Saúde Pública, Centro Integrativo e Muldisciplinar de Atendimento ao Portador de HTLV, Salvador, BA, Brasil.; 6 Universidade Federal do Pará, Instituto de Ciências Biológicas, Laboratório de Virologia, Belém, PA, Brasil.; 7 Imperial College Healthcare NHS Trust, St. Mary’s Hospital, National Centre for Human Retrovirology, London, United Kingdom.

**Keywords:** Human T-lymphotropic virus 1, Human T-lymphotropic virus 2, Diagnosis, Screening assays, Confirmatory assays, Algorithms

## Abstract

**INTRODUCTION:**

We present a data analysis and review of recent studies regarding the laboratory diagnosis of human T-lymphotropic virus 1 and 2 (HTLV-1/2) infections in Brazil.

**METHODS:**

Target populations, available diagnostic serological assays (screening and complementary tests), molecular assays (in-house), causes of false-positive and false-negative results, and flowcharts were analyzed.

**RESULTS:**

A table presents the target populations, two diagnostic flowcharts (depending on laboratory infrastructure and study population), and recent research that may improve how HTLV-1/2 is diagnosed in Brazil.

**CONCLUSIONS:**

Our results support the implementation of public policies to reduce HTLV-1/2 transmission and its associated diseases.

Human T-lymphotropic virus 1 (HTLV-1) was the first human virus described in the family *Retroviridae,* followed by HTLV-2. HTLV-1 and -2 share molecular and biological characteristics, such as the integration of viral nucleic acids (proviral DNA) into the cellular genome, establishing viral persistence, and maintaining and transmitting the virus. However, they differ in disease outcomes; HTLV-1 is responsible for at least two diseases with high morbidity and mortality, such as HTLV-1-associated myelopathy, and adult T-cell leukemia/lymphoma (ATL), whereas HTLV-2 seems to be less pathogenic. Thus, it is important to perform confirmatory and discriminatory diagnoses of HTLV-1/2 infections. 

Information on the prevalence of HTLV-1/2 infections in Brazil was recently published in the Epidemiological Bulletin of the Health Surveillance Secretariat, Ministry of Health^1^. Overall, it is estimated that 800,000 to 2.5 million people are living with HTLV-1/2 (PLHTLV), with prevalence varying by geographic region, individual ethnic background, education level, socioeconomic conditions, and risk factors. For instance, in a general population of Salvador, Bahia, a prevalence of 1.48% was reported^1^. Among blood donors, the prevalence of HTLV-1/2 varies from 0.03% to 0.48% and from 0 to 1.05% in pregnant women. Among all populations, a high prevalence was observed in the North and Northeast regions of Brazil and a lower prevalence in the Southern region^1^. 

Among vulnerable populations such as intravenous drug users; men who have sex with men; male and female sex workers; and people infected with other sexually transmitted diseases, mostly with human immunodeficiency virus (HIV), hepatitis B virus (HBV), and hepatitis C virus (HCV), high prevalence rates have been detected, varying from 0.7% to 5.3%^1^. Furthermore, the Epidemiological Bulletin highlights the current main transmission routes of HTLV acquisition as vertical (mostly by breastfeeding), sexual, and parenteral^1^. 

Identifying PLHTLV is essential as it enables multidisciplinary follow-up of patients and asymptomatic carriers and allows for the implementation of measures to prevent transmission, thereby reducing the medium- and long-term impacts to the Brazilian Unified Health System (*Sistema Único de Saúde, SUS*).

The serological diagnosis of HTLV-1/2 infections is based on the detection of virus-specific antibodies in biological fluids that are generated by an immune response directed against viral antigens encoded by structural and regulatory genes. Serological methods are classified into two categories: screening and complementary tests (confirmatory and discriminatory tests for HTLV-1/2 infections). Routine screening assays detect antibodies against HTLV-1/2; however, in addition to the probability of false-positives, screening assays do not differentiate between the two infections, thereby requiring confirmation of the results by highly specific tests that can distinguish infection by each viral type. Confirmatory molecular assays have been designed to identify segments of the proviral genome of HTLV-1/2 (proviral DNA) in mononuclear cells present in blood and cerebrospinal fluid, among other specimens.

HTLV serology became mandatory in blood banks in Brazil in 1993^2^ and in organ or tissue donors and recipients in 2009^3^. In 2016, HTLV confirmatory tests were incorporated into the *SUS* for individuals with ATL^4^. In other circumstances and populations, there are no national-level recommendations for the systematic use of serological screening tests or complementary assays for the diagnosis of HTLV-1/2 infections. Some Brazilian states have their own protocols for screening and confirmatory tests, relying on state and municipal resources. For instance, the states of Bahia, Minas Gerais, and Mato Grosso do Sul have antenatal HTLV screening programs.

Specific populations and contexts are associated with an increased risk of HTLV-1/2 infections. Therefore, testing these individuals is recommended (Table 1). Notably, a diagnosis of HTLV-1/2 enables adequate clinical management of the patient; disrupts mother-child, sexual, and blood transmission; and allows for counseling of infected individuals, thus improving quality of life and preventing new infections.

**TABLE 1:** Target populations and contexts for HTLV-1/2 testing in Brazil.

**Indications for HTLV-1/2 testing**
Individuals with clinical manifestations compatible with HTLV-1-associated diseases, such as uveitis, dermatitis, neurogenic bladder, Sjögren’s syndrome, and rheumatologic symptoms
Differential diagnosis of myelopathies
Blood, organ, tissue, and human milk donors
Organs and tissue recipients
Relatives and sexual partners of HTLV-1/2 carriers
Individuals with sexually transmitted and blood-borne infections
Pregnant women
Children exposed to HTLV
People who inject recreational drugs
Cases of occupational exposure to blood or biological material, such as accidents with sharp objects
Patients infected with *Strongyloides stercoralis*
Patients infected with *Mycobacterium tuberculosis*
Patients with leukemia or lymphoma

Abbreviations: **HTVL:** human T-lymphotropic virus.

Currently, third-generation or sandwich-type immunoenzimatic screening tests (enzyme immunoassay [EIA] or enzyme-linked immunosorbent assay [ELISA] and chemiluminescence (chemiluminescent immunoassay [CLIA]) tests are available in the national market. They contain synthetic peptides and/or recombinant or chimeric proteins of HTLV-1/2 as antigens and conjugates, and the conjugate is labeled with enzymes (EIAs) or acridinium (CLIAs). These have high sensitivity (close to 100%) and specificity (ranging from 92.0% to 99.5%), depending on the population and the employed test^5^ ^,^ ^6^. In low-risk populations such as blood donors, screening assays may have a low positive predictive value, as there is only a small chance of a reactive sample being a true positive. In this population group, the positive predictive values varied from 27.6% to 76.6%, depending on the EIA specificity and the assay used to confirm the HTLV infection: Western blot (WB) or line immunoassay (LIA)^1^. Therefore, complementary tests are necessary. Moreover, screening tests with low specificity (false positives) generate unnecessary costs for subsequent complementary tests and should be avoided^7^ ^,^ ^8^. To increase specificity and decrease screening costs, especially in populations and regions with a low prevalence of HTLV-1/2, serum pooling strategies were investigated at the Adolfo Lutz Institute of São Paulo^9^. The data indicated that serum pooling can reduce screening costs for *SUS* by more than 70%. In contrast, nonreactive results in routine screenings and true positives for HTLV-1/2 or for proteins produced by the HTLV genome may occur, although in small numbers, mostly when using reagents containing only recombinant proteins and/or synthetic peptides of the HTLV-1/2 viral envelope^5^ ^,^ ^10^. This was demonstrated by the description of HTLV-1 *Tax*-only carriers, who are seronegative in an HTLV-1/2 CLIA screening assay, a particle agglutination assay, and an in-house immunofluorescence assay on the MT-2 cell line^10^. 

The use of a chimeric antigen containing recombinant proteins from three regions of the proviral genomes of HTLV-1/2 (*env* [rgp46], *gag* [p19], and *pX* [Tax]) may increase the sensitivity of screening tests (unpublished data; studies conducted at the Federal University of Minas Gerais and Hemominas Foundation, Belo Horizonte, Minas Gerais). In addition, chimeric antigen for each type of HTLV may reduce false-positive results due to the absence of p24 antigens in individuals who are truly infected with HTLV-1^11^. Notably, a rapid screening test that detects all HTLV strains circulating in Brazil is needed. Recently, a recombinant multi-epitope antigen was developed in Brazil for this purpose (patent BR1020180123700). 

The development of a point-of-care serological test with high sensitivity and specificity is of global urgency, as highlighted in the 2021 HTLV technical report issued by the World Health Organization^12^. This would facilitate epidemiological studies that include difficult-to-assess populations due to geographic conditions or social vulnerability. These tests would also be useful for antenatal screening, where a rapid turnaround time is essential for implementation of measures to prevent new infections. Point-of-care tests, also known as rapid tests, allow for diagnosis outside of the laboratory environment and are included in the *SUS* healthcare routines. Rapid tests for HIV-1, *Treponema pallidum* (syphilis), and HBV and HCV have already been implemented by the Brazilian Ministry of Health^13^.

There are two types of complementary (confirmatory and discriminatory tests for HTLV-1/2 infections) tests: serological and molecular. Serological complementary tests examine the presence of antibodies against specific HTLV-1/2 antigens. The tests available in the national market include the WB assay, which uses nitrocellulose strips containing HTLV-1 whole viral lysate and recombinant HTLV-1 (rgp 46-I), HTLV-2 (rgp 46-II) envelope proteins, and the transmembrane glycoprotein common to HTLV-1/2 (GD 21), and the LIA, which identifies synthetic peptides and recombinant proteins common to both viruses and specific to each virus adsorbed on nylon strips. These complementary tests have different sensitivities and can generate inconclusive results (untyped HTLV and indeterminate), especially when applied to samples obtained from individuals with HTLV-2 infections who are coinfected with HIV, HBV, or HCV. In these populations, the LIA sensitivity varied from 94.8% to 97.2% and the specificity was approximately 82.0%, while the WB sensitivity varied from 82.4% to 82.8% with a specificity of approximately 60.0%; thus, in these cases, LIA is the test of choice^5^ ^,^ ^14^. 

Inconclusive results in complementary serological tests may be associated with different factors, including (1) the period of seroconversion, (2) stringent manufacturer positivity criteria, (3) HTLV strains distinct from those used in the tests, (4) the presence of defective viral particles, and (5) point mutations in the long terminal repeat viral promoter region and regions that encode structural and regulatory proteins, mainly in the HTLV-2 viral envelope, among others^5^ ^,^ ^11^ ^,^ ^14^ ^-^ ^16^.

Complementary tests using molecular techniques can identify the proviral DNA of HTLV-1/2 because HTLV maintains infection mainly by clonal expansion and the presence of viral RNA is extremely rare in the plasma. These tests are based on polymerase chain reaction (PCR) in various formats: nested PCR, PCR followed by a restriction enzyme site search resulting in fragments of different sizes (restriction fragment polymorphism analysis), real-time or quantitative PCR (qPCR), and loop-mediated isothermal amplification (LAMP).

In all molecular assays, oligonucleotides (primers and/or probes) specific to conserved regions of the proviral genome of HTLV-1/2 (mainly the *pol* and *tax* segments) were used. In Brazil, commercial assays are not available, only in-house assays, many of which have not been validated. There is no consensus on which test has the best performance, especially for HTLV-2 strains that circulate in Brazil. Moreover, depending on the study population, positivity percentages differ from more than 95% for individuals with HTLV-1 monoinfection and close to 60% for the HIV-coinfected population^5^ ^,^ ^17^ ^-^ ^20^. False-negative results in molecular complementary assays may be due to low-quality extracted DNA, low proviral load, the presence of defective proviral particles, and point mutations^16^. However, due to the lower cost of confirmatory molecular tests compared to complementary serological tests (LIA and WB), its use is recommended as the first option, followed by serological tests for samples with negative PCR results. This strategy aims to reduce the cost of HTLV diagnosis for the *SUS* (from 39% to 70%, depending on the population studied)^5^ ^,^ ^20^. 

Two approaches have recently been proposed to reduce the cost of an HTLV-1/2 diagnosis confirmed by molecular tests. The first approach is a multiplex qPCR, which was developed by researchers from the Adolfo Lutz Institute in São Paulo for use in an open platform with several brands of reagents available in the national market (https://pesquisa.bvsalud.org/ses/resource/pt/biblio-1051598). This approach differs from that described previously in Brazil, which was standardized using only one master mix reagent and one specific type of equipment^21^. The second approach is based on the LAMP techinique^22^. Both approaches are good options. The former employs only one qPCR assay and detects HTLV-1/2 and endogenous genes in a single test; the latter uses more primers and two test tubes (one for HTLV-1 and another for HTLV-2) but does not require sophisticated equipment (only a water bath or heating block) making this technique useful in contexts of limited resources. Although these techniques have been validated using samples from monoinfected and coinfected individuals, a national study is needed to confirm their efficiency, as with all new screening assays (rapid tests and EIAs with chimeric antigens).

qPCR assays can be used for both the diagnosis and monitoring of proviral loads in patients with symptoms related mainly to HTLV-1 infection^18^ ^,^ ^19^ ^,^ ^21^. The use of synthetic nucleotides or plasmids to build standard curves and to determine the proviral load of HTLV-1 has been indicated as a feasible, low-cost alternative to using cell lines^23^.

Regarding an HTLV diagnostic flowchart applicable to Brazil, laboratories without a molecular biology infrastructure can confirm results using the WB or LIA serological tests, preferably LIA due to the higher sensitivity shown for HTLV-1 monoinfection and HTLV-1/2 coinfections with other viruses^14^. Notably, in these cases, LIA was capable of confirming HTLV-1/2 infections in 61% of WB indeterminate samples from patients with HIV and in 83.4% of samples from patients with HBV and HCV from São Paulo. LIA also confirmed HTLV-1 infections in 76% of WB indeterminate samples from an HTLV outpatient clinic in Salvador, Bahia^14^. This proposal was recently corroborated by a study in Japan; the WB was replaced with LIA in the diagnostic flowchart for HTLV-1 infection^24^. 

When samples have inconclusive results in complementary serological tests and the laboratory is not able to perform molecular assays, it is recommended to retest the patient after three months, as the first test may have been conducted during the period of seroconversion (Figure 1A). If the patient presents with symptoms associated with HTLV-1, they should be referred to a laboratory that performs molecular assays. For laboratories that have infrastructure and supplies to perform both complementary tests (serological and molecular) and are attempting to achieve a better cost/benefit ratio, molecular tests should be the first option (Figure 1B). In the case of discordant results between screening and molecular tests, confirmatory serological tests should be performed, with LIA being the first choice^5^ ^,^ ^14^ ^,^ ^24^. 

**FIGURE 1:**
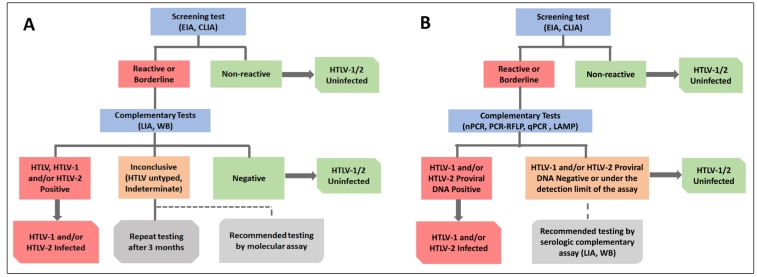
Flowcharts for HTLV-1 and HTLV-2 diagnosis in Brazil using serological complementary tests (**A**) or molecular complementary tests (**B**).

CLIA: chemiluminescence immunoassay; EIA: enzyme immunoassay; HTVL: human T-lymphotropic virus; LAMP: loop-mediated isothermal amplification; LIA: line immunoassay; PCR-RFLP: polymerase chain reaction-restriction fragment length polymorphism; qPCR: quantitative real-time polymerase chain reaction; WB: western blot. Complementary tests were confirmatory and discriminatory tests.

These flowcharts apply to the general population, pregnant women, and children over 18 months of age if they are not breastfeeding. Pregnancy does not interfere with the detection of anti-HTLV antibodies or with the detection of HTLV-1 proviral genome segments^25^; maternal antibodies passively acquired during pregnancy degrade after 18 months and those that remain are due to seroconversion to HTLV^26^.

Although there are gaps, the current Brazilian experience with HTLV diagnosis can provide support for health authorities and managers in the implementation of reliable tests for the surveillance and diagnosis of HTLV-1/2. This may help to reduce transmission, provide adequate treatment and care for PLHTLV, and implement compulsory notification of HTLV-1/2 infections. A nationwide multicenter validation study of in-house molecular tests is important and should be considered a priority for improving the laboratory diagnosis of HTLV in Brazil.
